# The complete chloroplast genome sequence of a *Castanea henryi* cultivar

**DOI:** 10.1080/23802359.2019.1698376

**Published:** 2019-12-17

**Authors:** Ying-Lin Li, Guang-Shi Gu, Jun-Jian Wu, Bin Liu, Shu-Tao Ye, Hui Chen, Shi-Pin Chen, Yu Li

**Affiliations:** College of Forestry, Fujian Agriculture and Forestry University, Fuzhou, PR China

**Keywords:** *Castanea henryi*, chloroplast genome, phylogeny, fagaceae

## Abstract

*Castanea henryi*（Skam）Rehd. et Wils is an important woody plant producing nuts in China. In this study, the complete chloroplast genome sequence of *C. henryi* was reported by using the Illumina Hiseq 2500 platform. The complete chloroplast sequence is 160,807 bp, including large single-copy (LSC) region of 90,394 bp, small single-copy (SSC) region of 18,963 bp, and a pair of invert repeats (IR) regions of 25,725 bp. Plastid genome contains 112 genes, 78 protein-coding genes, 30 tRNA genes, and 4 rRNA genes. Based on 26 chloroplast genomes, phylogenetic analysis indicates that *C. henryi* is closely related to *C. mollissima* in Fagaceae.

*Castanea henryi* (Skam) Rehd. et Wils belongs to Fagaceae. It is widely distributed in hills and mountains, which are lower than 1800 m above sea level at the middle subtropical zone. It is a tree species of the genus *Castanea*, which is a famous woody grain and fruit tree species in southern China. The nuts are sweet and delicious, and the flavor is better than that of other chestnut. Besides, *C henryi* has a series of excellent characteristics such as straight trunk, high resistance to stress, high yield (Fan et al. 2015). As a unique chestnut plant in China, it is now widely cultivated in the mountainous areas of northern Fujian province and southern Zhejiang province (Chen 2000). Over the past few decades, the farmers and breeders have selected a number of cultivars with good yield and quality (Li et al. 2018).

The material selected in this study is the excellent cultivars “Jianou No.1”, which selected by the breeders from College of Forestry, Fujian Agriculture and Forestry University. The “Jianou No.1” is located in Jianou City, Fujian Province (26°38′N, 117°57′E).

DNA was extracted from fresh leaf tissue, with 500 bp randomly interrupted by the Covaris ultrasonic breaker for library construction. The paired-end library was sequenced by Illumina Hiseq 2500 platform, approximately 2GB data generated. Illumina data were filtered by script in the cluster (default parameter: -L 5, -p 0.5, -N 0.1). Complete plastid genome of *C. mollissima* (GeneBank accession: HQ336406) as a reference, plastid genome of *C. henryi* was assembled by GetOrganelle pipe-line (https://github.com/Kinggerm/GetOrganelle). It can get the plastid-like reads, and the reads were viewed and edited by Bandage (Wick et al. 2015). Assembled choroplast genome annotation was based on comparison with *C. mollissima* by Geneious v 11.1.5 (Biomatters Ltd., Auckland, New Zealand) (Kearse et al. 2012). The annotation result was draw with the online tool OGDRAW (http://ogdraw.mpimp-golm.mpg.de/) (Lohse et al. 2013).

The complete plastid genome sequence of *C henryi* (GenBank accession: KX954615) was 160,807 bp in length, with a large single-copy (LSC) region of 90,394 bp, a small single-copy (SSC) region of 18,963 bp, and a pair of inverted repeats (IR) regions of 25,725 bp. Complete chloroplastid genome contain 112 genes, and there were 78 protein-coding genes, 30 tRNA genes, and 4 rRNA genes. The complete genome GC content was 36.75%. In order to reveal the phylogenetic position of *C. henryi* with other members of Fagaceae, a phylogenetic analysis was performed based on 24 complete chloroplast genomes of Fagaceae, and two taxa (*Alnus cordata*, *Betula cordifolia*) as outgroups. The chloroplast genomes were downloaded from NCBI GenBank. The sequences were aligned by MAFFT v7.307 (Katoh and Standley 2013), and phylogenetic tree was constructed by RAxML (Stamatakis 2014). The phylogenetic tree showed that *C. henryi* was most closely related to *C. mollissima* (Figure 1).

**Figure 1.**
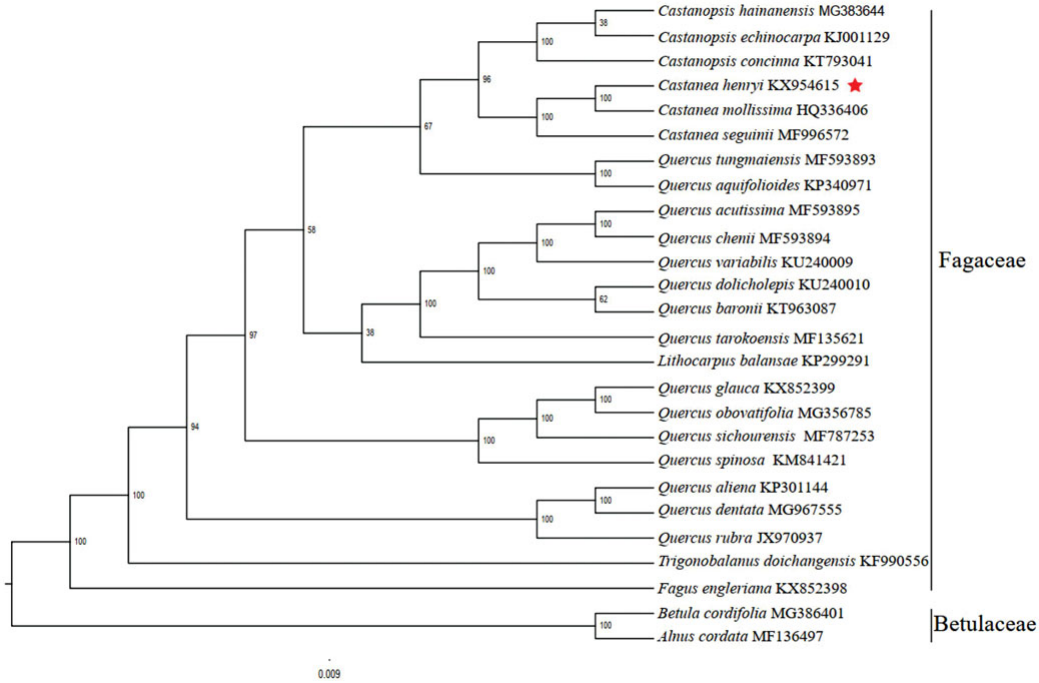
Phylogenetic analysis of 24 species of Fagaceae and two taxa (*Alnus cordata*, *Betula cordifolia*) as outgroup based on plastid genome sequences by RAxML, bootstrap support value near the branch.
